# Comparison of the effects of 2 frequencies of application of photobiomodulation on facial rejuvenation: Controlled, randomized, and double-blind clinical trial

**DOI:** 10.1097/MD.0000000000032514

**Published:** 2023-02-03

**Authors:** Erick Frank Bragato, Jefferson André Pires, Marcos Momolli, Marina Bertoni Guerra, Adriana Fernandes Paisano, Raquel Agnelli Mesquita Ferrari, Sandra Kalil Bussadori, Lara Jansiski Motta, Kristianne Porta Santos Fernandes

**Affiliations:** a Postgraduate Program in Biophotonics Applied to Health Sciences, Universidade Nove de Julho (UNINOVE), São Paulo, Brazil; b Postgraduate Program in Rehabilitation Sciences, Universidade Nove de Julho (UNINOVE), São Paulo, Brazil.

**Keywords:** light emitting diode (LED), optical coherence tomography (OCT), photobiomodulation (PBM), skin rejuvenation

## Abstract

Skin aging is an irreversible, slow and progressive process, mainly influenced by age, but also by external factors such as ultraviolet radiation, smoking, and alcohol, among others. It is increasingly common to look for procedures that slow down skin aging by limiting or hiding its effects on appearance. Studies have shown the benefits of photobiomodulation (PBM) for the skin, especially with the use of red light-emitting diodes. However, there is a high level of variability in the treatment parameters and frequency of application.

**Methods and analysis:**

The objective of this study is to compare the effects of PBM with a light-emitting diode mask (660 nm, 6.4 mW/ cm², 8,02 J/ cm², 5.02 mW, 21 minutes) on facial rejuvenation using 2 frequency applications for 4 weeks: one group will receive PBM application on the face, twice a week and another group will receive PBM application 3 times a week. A group with simulated PBM applied twice a week for 4 weeks will be used as a control. The treatment will be performed on female participants aged between 45 and 60 years. After 4 weeks, evaluations of photographic images by specialists (Wrinkle Assessment Scale) as well as the quantitative analysis of the wrinkle size by the Image J software, the depth and width of wrinkles (assessment of face impressions by optical coherence tomography) and the level of Satisfaction with Facial Appearance Overall will be compared with data collected before the start of the study. All data will be analyzed statistically according to their distribution, seeking a level of statistical significance of 0.05.

**Ethics and dissemination:**

This protocol was approved by the Research Ethics Committee of the Nove de Julho University (acceptance number: 4.365.565). This trial has been registered in ClinicalTrials.gov (ID: NCT04911140). This study is recruiting.

## 1. Introduction

Human skin aging results from the combination of biochemical, morphological, and physiological changes over time, genetic and epigenetic factors, and cumulative damage caused by external factors, including smoking, diet, severity, and most notably, ultraviolet radiation.^[1–6]^ These changes decrease the musculoskeletal support of the face, thus leading to loss of volume and changes in shape that can impact the quality of life, social relationships, and activities of individuals. Thus, preventive and corrective interventions are closely related not only to health but also to psychosocial well-being.^[7–9]^

The search for procedures to slow the aging process is increasing; limiting or hiding its effects on the skin and its appearance. The main surgical procedures for facial rejuvenation are facelift, rhinoplasty, otoplasty, and blepharoplasty. Facial lifting aims to improve facial and cervical aesthetics by suspending muscle compartments and skin giving the patient a more youthful appearance.^[10]^

In recent years, both dermatology and plastic surgery have given increasing importance to minimally invasive rejuvenation treatments capable of providing satisfactory results.^[11,12]^ However, even though they are minimally invasive, these procedures are not without complications, which can involve not only local pain, edema, and ecchymosis.^[13]^

On the other hand, photobiomodulation (PBM), which has also been used in facial rejuvenation, consists of the use of non-ionizing, non-thermal, and non-ablative light, without side effects and is capable of generating analgesic, anti-inflammatory and repairing results.^[14,15]^ PBM most commonly employs light (light amplification by stimulated emission of radiation [LASER] and light emitting diode [LED] devices) in the range of visible red and near-infrared (600–1100nm) to achieve desired therapeutic effects.^[16]^ Dosimetric parameters of PBM include wavelength, radiant energy and power, irradiance, and radiant exposure which are directly related to the ability of light to penetrate tissues and produce biological effects.^[14]^

The therapeutic effect of LEDs can be as efficient as that of LASERs as long as the same dosimetric application parameters are respected. These sources have advantages in the fact that they can be grouped for applications in larger areas and still bring security for home use.^[14,17,18]^

As previously mentioned, some studies have been evaluating the effects of PBM on facial rejuvenation, with the majority using red light.^[19–26]^ However, there is a high level of variability in the treatment parameters, in the frequency and duration of the sessions, and in the total time of application of the PBM, hence the need for controlled studies to define the ideal parameters. This study was concerned with comparing the effects of 2 regimes of application of PBM on facial rejuvenation.

## 2. Materials and methods

### 2.1. Study design

This randomized, controlled, blinded clinical trial was written according to the Standard Protocol Items: Recommendations for Interventional Trials checklist and following the Declaration of Helsinki. This protocol was approved by the Research Ethics Committee of the Nove de Julho University on October 27, 2020 (acceptance number: 4.365.565). This trial has been registered in ClinicalTrials.gov (ID: NCT04911140). This study is recruiting.

After a verbal and written explanation of the study, the participants who agree to take part in the research will sign the informed consent form. All participants will be seen at the clinic of specialties at Nove de Julho University - São Paulo, Brazil.

**Figure 1.**
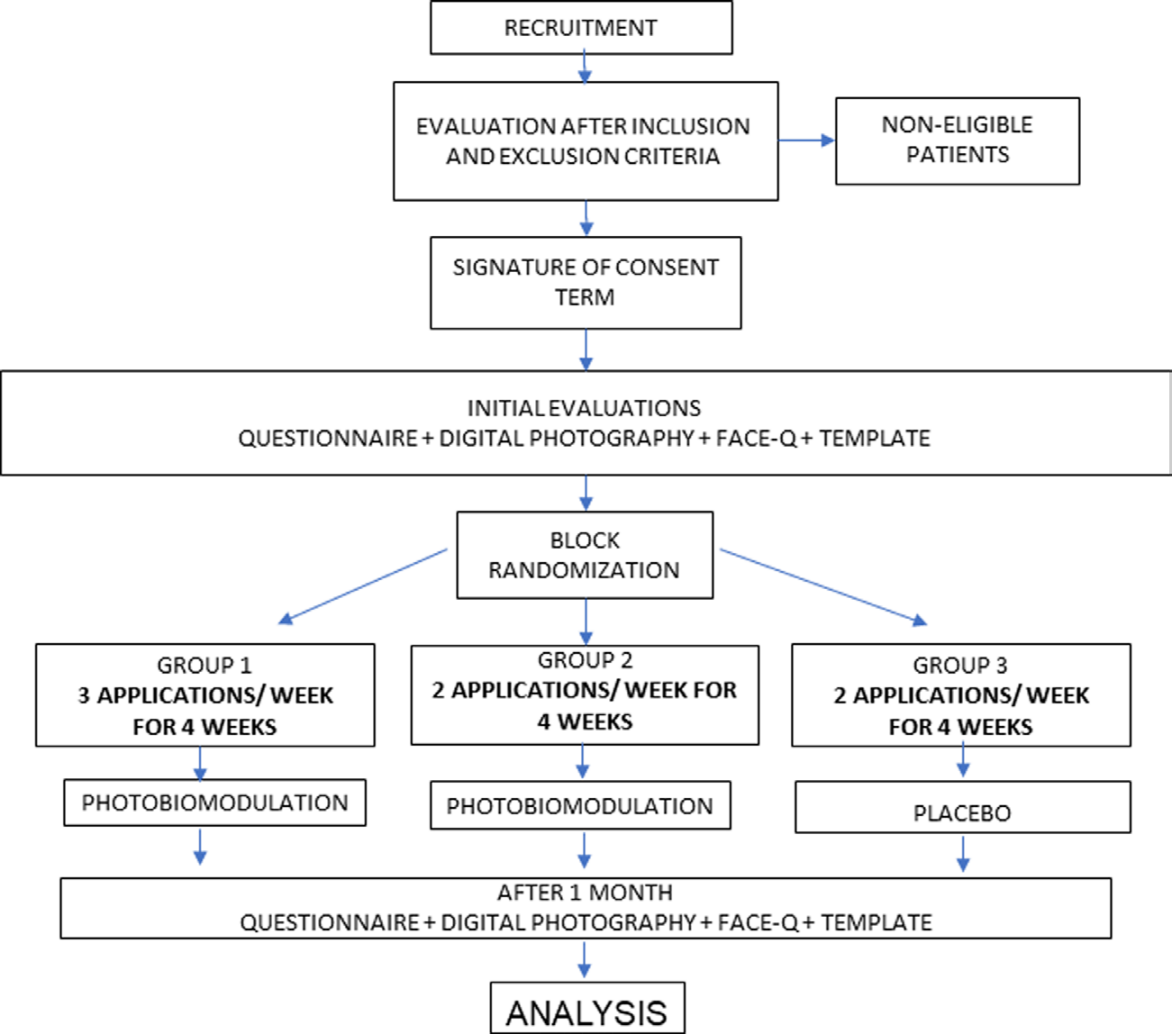
SPIRIT schedule of enrollment, interventions, and assessments. Pg 5. SPIRIT = Standard Protocol Items: Recommendations for Interventional Trials.

The patients and/or the public were not involved in the design, recruitment, and conduct of the study. Standard Protocol Items: Recommendations for Interventional Trials schedule of enrollment, interventions, and assessments according to Figure 1.

#### 2.1.1. Inclusion criteria.

A screening questionnaire with identification information of the participant will be applied, such as name, age, address, telephone, email, marital status, and education, in addition to a brief anamnesis on comorbidities, medications in use, and procedures performed on the face, among others. Only participants who meet the eligibility criteria will be included in the study such as female volunteers, aged 45 to 60 years, at menopause (at least 12 months without menstruation), and healthy.

In the same questionnaire, participants will be classified according to the skin phototype by the Fitzpatrick classification.^[27]^ The phototype of each participant will be identified, and only those that have a score will participate in the research, that is, phototypes from I to IV. The Glogau classification^[28]^ will also be part of the questionnaire, a categorization system for photo-damaged skin that estimates the level of skin damage. This system has been useful to assess levels of the dermo-epidermal lesion and fits the skin into four types. Only participants categorized as Glogau type II to IV will be part of the study.

#### 2.1.2. Exclusion criteria.

Participants that will be excluded from the study:

With a history of photosensitivity.
Who has any type of lesion on the skin on the face.
Who use corticosteroids, anticoagulants, or any drug known to increase photosensitivity, including systemic retinoids and use of topical retinoic acid in the last 6 months.
People with any collagen-related diseases, malnutrition, anemia, immunosuppression, cancer diseases, smokers, predisposition to hypertrophic and keloid scarring, history of dermatological diseases, surgery on the face, trauma on the face, diseases that could affect the condition of the skin, and psychiatric disease.
Who are in the menstrual period, in the climacteric, or in hormone replacement therapy.
Who underwent cosmetic procedures on the face, such as application of botulinum toxin in the last 8 months, facial filling in the last year, chemical peels, ablative LASER, and dermabrasion in the last 6 months.
Who does not respect the post-treatment recommendations or fails to attend a treatment session.
Patients who present an adverse event (hypersensitivity, allergies) will not be part of the statistical analysis. However, these data will be described and discussed, as well as the possible adverse effects and the participants will receive treatment to resolve the condition. These data will be described and treated according to the complication.

#### 2.1.3. Sample size calculation.

The sample size was calculated based on the primary outcome. The outcome considered was the classification of the Lemperle Wrinkle Assessment Scale (WAS).^[29]^ In the study by Sylvie Boisnic,^[30]^ the authors observed that the Lemperle score decreased in both groups after 12 weeks of treatment with a reduction of 1 (±0.7) point in the experimental group and 0.34 (±0.5) point in the placebo group and a statistically significant (*P* < .0002) change from baseline between the 2 groups after 8 weeks of treatment. This study was designed to detect a difference in scores between the groups, considering the mean and standard deviation of the study by Sylvie Boisnic,^[30]^ for the analysis of variance (ANOVA) with 3 groups and repeated measures, with significance level of 5% and power of 95%, with equal allocation for the three groups, requiring 24 participants per group, 72 in total. To control a possible loss, 50% can be added to the sample, with 36 participants per group, that is, 108 in total. The sample calculation was performed using the G-Power Version 3.1.9.4 program (Universität Kiel, Germany).

#### 2.1.4. Randomization.

After completing a questionnaire and being evaluated by the researcher for inclusion and exclusion criteria, a person not involved in the study will randomly distribute the participants among the three experimental groups using an online random sequence generator program (https://www.randomizer.org/tutorial/). After this, opaque envelopes, containing the generated number, will be sealed and will remain in a safe place until participants are recruited for the outpatient clinic. When the participant enters the clinic, the researcher will open the corresponding envelope and begin the specified treatment. Participants will be divided into 3 groups:

(Group 1) Application of PBM twice a week for 4 weeks;

(Group 2) Application of PBM three times a week for 4 weeks;

(Group 3) Application of simulated PBM twice a week for 4 weeks;

#### 2.1.5. Blinding procedures.

The study will be carried out by a research team that will include a researcher who will carry out the application, undergraduate students, and 3 plastic surgeons who will carry out the initial evaluation of the photographs and the reevaluations after the application of the treatment (blind as to the composition of the groups), and a statistician for data processing.

The researcher responsible for the PBM application will pick and open the envelope containing the information of the experimental group in which the participant will be inserted and will proceed with the experiment. A single researcher will carry out the treatment and it will not carry out any type of evaluation. Pre and post-treatment assessments will be made by 3 examiners (3 plastic surgeons) who will not be aware of the group to which each patient will be allocated. Participants will not be aware of whether or not they have received the application of the PBM, as they will be using dark goggles. The person responsible for the application will position the equipment in the irradiation locations in all participants and will only activate the light when provided for in the specific experimental group. In the control group, the mask will be installed and kept off and a white light will be turned on close to the participant’s face so that they have a sensation of luminosity even using the blindfold. The characteristic sound of the device will be triggered by recording in the placebo group for the PBM.

#### 2.1.6. Interventions.

The participants will be instructed not to use any cream with an active element for 2 months. All of them will receive a bottle of neutral hydrating cream for daily use in the morning and before bed. The cream formulation is composed of carbomer, cetearyl alcohol, glyceryl stearate/polyethylene glycol-100 stearate, helianthus annuus seed oil, butylated hydroxytoluene, disodium ethylenediaminetetraacetic acid, glycerin, propylene glycol, aminomethyl propanol, phenoxyethanol, ethylparaben, butylparaben, propylparaben, isobutylparaben, and aqua.

Each participant will have their face cleaned with a neutral nonalcoholic wet wipe and their eyes blindfolded and protected with goggles that allow the rest of the face to be illuminated with the red LED or with simulated light without the participant knowing which treatment was applied. A transparent plastic film will be used to cover the face before placing the LED mask, material supplied by the same manufacturer of the mask.

**Figure 2.**
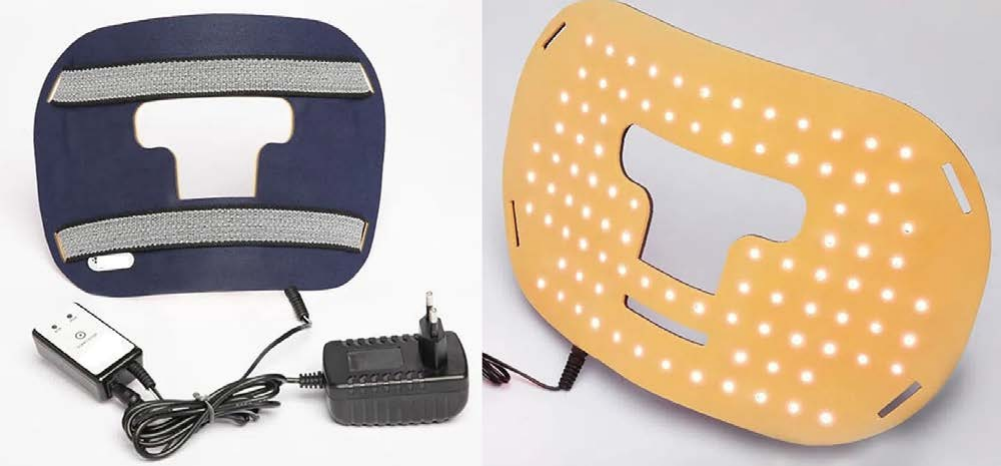
LED facial mask, Cicatrillux Bionext (Cosmedical, Mauá, São Paulo—Brazil). Pg 9. LED = light emitting diode.

The equipment used will be LED masks (Cicatrillux Bio next from Cosmedical, Mauá, São Paulo—SP, Brazil) (Fig. 2), containing 92 LEDs with red wavelength (660 ± 10 nm) in the parameters described in Table 1. The application time will be 21 minutes per session, totaling 3 cycles of 7 sequential minutes due to the device’s shutdown time. The LED mask will be applied 3 times a week for 4 weeks in patients in group 1 and twice a week for 4 weeks in patients in group 2. In participants in group 3, the mask will be kept off and a white light next to the stretcher will be turned on for the participants to have the sensation of luminosity. The same beep will be emitted each cycle and the participants received the simulated application twice a week for 4 weeks.

**Table 1:** LED device dosimetric parameters. Pg 10.

Parameters	Red LED
Wavelength (nm)	660 (±10 nm)
Spectral bandwidth (nm)	20
Operation mode	Continuous
The radiant power of each LED (mW)	5.02
Polarization	Random
Opening diameter of each LED (mm)	10
Irradiance at the opening of each LED (mW/cm^2^)	6.4
Beam area on target (cm^2^)	0.785
Application time (min)	21
Radiant LED exposure (J/cm^2^)	8.02
Radiant energy by LED (J)	6.3
Light emission angle	90º
Application mode	Contact
Anatomical location of application points	Face

LED = light emitting diode.

### 2.2. Variables of the study

The outcomes of the study will be the evaluation of wrinkles by independent observers; analysis of the width and depth of wrinkles; wrinkle length by Image J software (National Institutes of Health, Bethesda, Maryland, https://imagej.nih.gov/ij/download.html); and assessment of satisfaction with facial appearance by participants.

#### 2.2.1. WAS.

The evaluation of wrinkles will be carried out in a standardized way using the WAS.^[29]^ This scale was developed and validated to quantify facial wrinkles based on predefined photographic models. To take photographs of the face, the participants will sign the image authorization term for use in the research and must be without any type of makeup. In a specific and reserved room, digitalized photographs of the face will be taken in the positions: front and 45 degrees right and left; with a Canon digital camera model SX510 HS 12.1 MP (Canon Do Brasil Indústria E Comércio | Ltda. São Paulo, SP, Brazil), using white LED lighting, turquoise blue background, 50 cm face distance, manual macro mode, a standard that will be followed in the photographs 30 days after the end of the treatment. The images obtained will be edited and standardized to be evaluated by the specialists according to the WAS.^[29]^ All images will be stored on a virtual disk which only the researcher will have access using a password. The images will be distributed later to the 3 plastic surgeons who will classify them according to the scale of wrinkles and photographic models pre-defined. Each evaluator will receive the same image of wrinkles, all at the end of the experiment. Each image should receive a score from 0 to 5 using the WAS as described previously.^[29,31]^ The data obtained will be grouped and the evolutionary comparison will be made according to the group of the patient under study.

To ensure homogeneity in the evaluation, an intra and interobserver agreement calculation will be performed before the sample is evaluated. Each specialist will receive 20 photographs of patients who did not participate in the study to classify facial wrinkles at any given time. After 7 days they will receive the same photographs and will need to classify the wrinkles again. The agreement index will be calculated for each evaluator and between the evaluators and an acceptable value of agreement must be above 70%.

#### 2.2.2. Analysis of the width and depth of wrinkles by optical coherence tomography (OCT).

**Figure 3.**
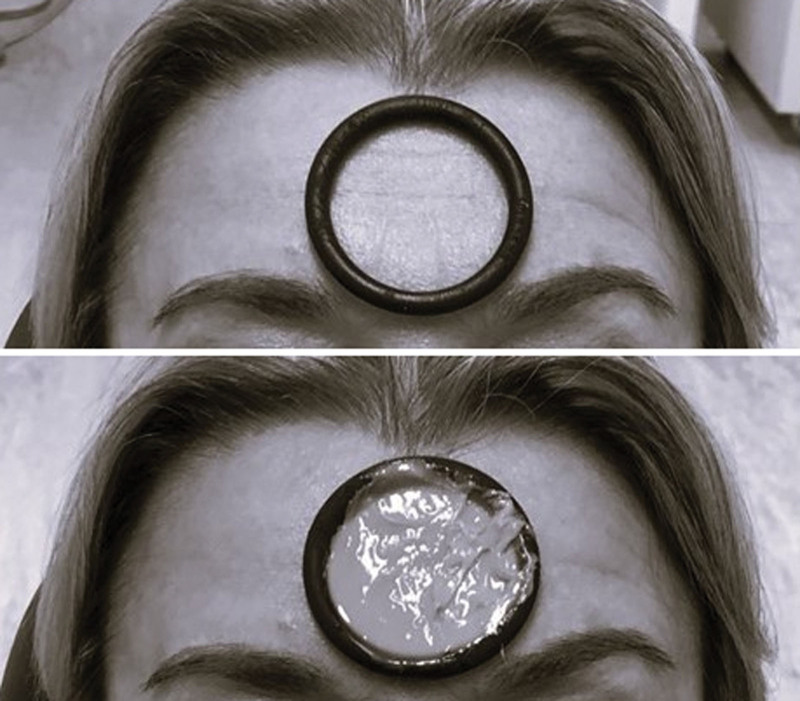
Face impression with condensation silicone. Pg 11.

**Figure 4.**
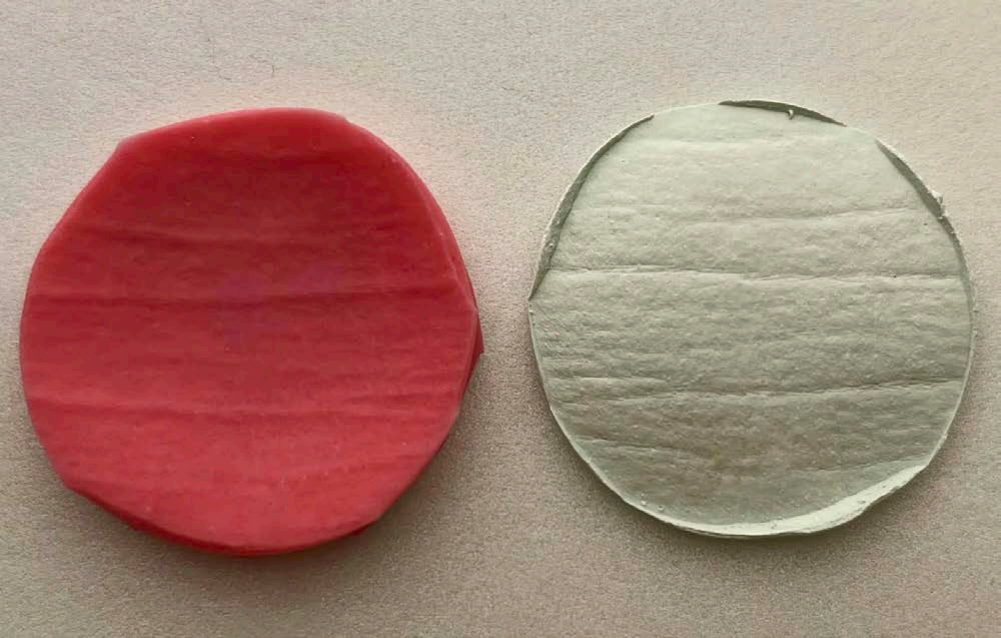
Silicone and plaster casts for optical coherence tomography analysis. Pg 11.

Before treatment and after 1 month, molds of the frontal lines will be made with light condensation silicone (Clonage, DFL Indústria e Comércio, RJ, Brazil). The silicone mold obtained will be poured into special plaster in a short time. Plaster models will be sent for OCT analysis and wrinkle width and depth measured before treatment and after 1 month. The models will be made of a special type of plaster, which has greater resistance and fidelity in the reproduction of details when compared to other types (Fig. 3). Two impressions were taken for each patient, an initial one, before the application of PBM, and a final one, after the treatment was completed. The region selected for the study will be the frontal area, above the glabella. Patients will be instructed to remain in the lying position so that the impression is performed (Fig. 4).

**Figure 5.**
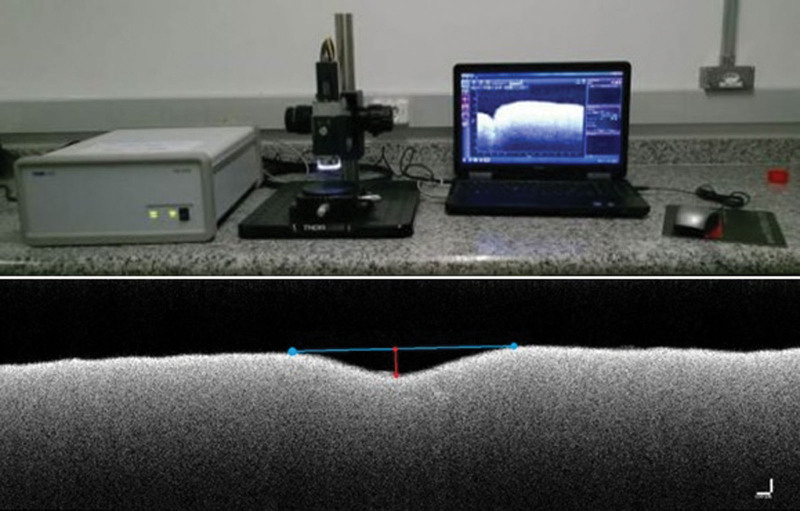
Image of the wrinkle impression obtained by OCT. Pg 12. OCT = optical coherence tomography.

The OCT-CALLISTO tomograph from the Multiuser Center of the Federal University of ABC will be used to obtain the images by OCT. The plaster models obtained before and after the treatment will be evaluated with the technique. First, a visual assessment of the molds will be performed to select a visible wrinkle. The wrinkle position for analysis will be flagged to ensure that the same wrinkle and region will be analyzed before and after treatment. From the selected point, an image will be obtained with the OCT technique (Fig. 5). The images will be analyzed using a computational routine in Python language. Ten equidistant measurements of the width and depth of the wrinkle will be performed and the value obtained for calculation purposes will be the average of these measurements.

#### 2.2.3. Quantitative evaluation of wrinkles.

The same images obtained for expert evaluation will be analyzed by the Image J software to quantify wrinkles. They will be quantified by the variation of pixels in the image of frontal wrinkles, periorbital, glabella, and nasolabial folds before and after 1 month of treatment. The ImageJ software analyzes the image by the intensity or gray level of the pixels. The calculation of the areas is done by counting the pixels of the selected regions.^[32]^ A researcher who was not involved in the study will take the measurements and will not know which treatment group the patient belongs to. Therefore, the evaluator will be blind regarding the group but will need to know which image was the before and after to compare the measurements of the same wrinkles.

#### 2.2.4. Assessment of the satisfaction with facial appearance.

Participants will answer the Satisfaction with Facial Appearance Overall (FACE-Q) questionnaire (adapted for the Portuguese language, before treatments and after a month). FACE-Q is a tool developed to measure the impact and effectiveness of facial aesthetic procedures from the patient’s perspective. The results will be tabulated and evaluated according to the sum of the grades obtained.^[4,33,34]^

### 2.3. Statistical analyses and data analyses plan

**Figure 6.**
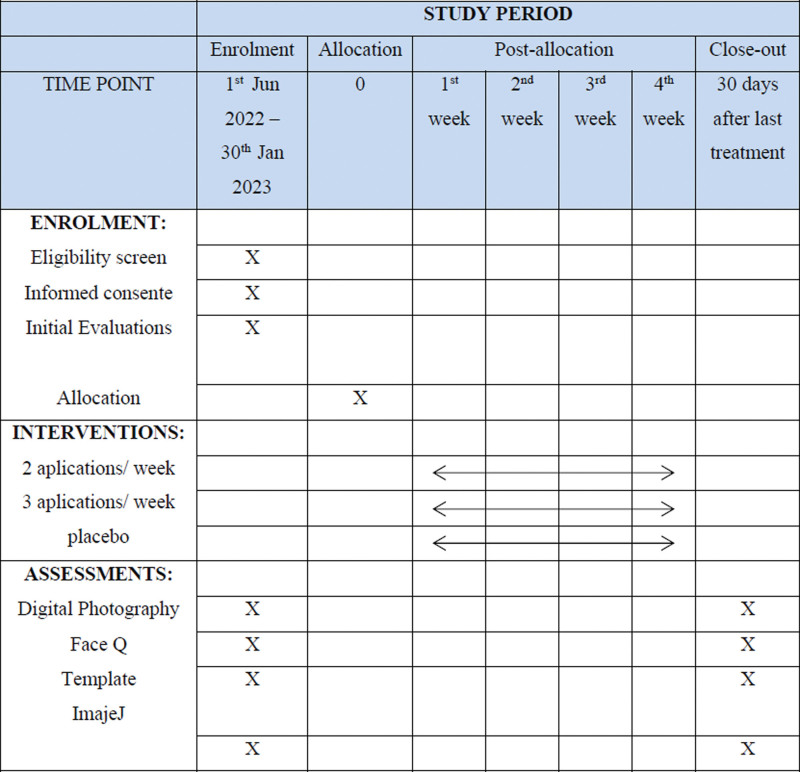
Flowchart activity – study timeline. Pg 13.

The criteria of normality and homogeneity of variances will be verified by ANOVA, normality will be verified with the Shapiro–Wilk and Kolmogorov–Smirnov tests. The chi-square test will be used to compare groups for categorical variables. Numerical variables will be compared by repeated measures ANOVA. If there is a statistical difference between the groups, Tukey post hoc test will be used. In all tests, a significance level of 0.05 will be adopted, which will result in an analysis with a 95% confidence interval. The software that can be used is SPSS 23 (IBM, New York) and Statistica 8.0 (StatSoft, TIBCO Software Inc, CA). The complete study schedule is shown in Figure 6.

## 3. Discussion

PBM, through the use of LEDs and LASERs, has shown good results in the treatment of wounds, increasing the formation of fibroblast cells, collagen synthesis, and reduction and control of inflammatory cells with a consequent increase in tissue formation.^[35]^ Recently, some studies have proposed the use of PBM for aesthetic medicine.^[36]^ The use of LED devices brings practicality and safety to applications and has also been evaluated for facial rejuvenation, mainly in the red wavelength.^[17]^ PBM is indicated for mild facial photoaging, fine wrinkles, skin damage, and texture changes. They are not indicated as replacement therapy for loss of volume, facial contour, sagging skin, or deep rhytids.^[25]^ However, dosimetric parameters, application frequencies, and total treatment time vary greatly.

In addition, the instruments used to evaluate the results are often subjective, highlighting the need for additional evaluations through more precise methodologies. This work described a randomized, controlled, double-blind clinical trial protocol based on the comparison of a treatment to be performed in 3 groups for 4 weeks: one group will receive the application of the red LED mask 3 times a week, another group will receive treatment 2 times a week and the control group will perform the simulated application of the LED mask twice a week. To increase participant compliance, a neutral face cream formulation was donated to be used by all patients at night during treatment.

The sample should be as homogeneous as possible to minimize individual biases, hence the importance of delimiting the age of participants between 45 and 60 years old, Glogau from 2 to 4, and who are in menopause. It is known that menopausal hypoestrogenism leads to changes in skin metabolism, such as changes in collagen fibers and reduced water content, favoring aging.^[37]^ Skin types II, III, and IV were chosen as inclusion criteria to standardize the melanin content of the participants, excluding the other phototypes I, V, and VI to reduce light absorption by melanin.^[38]^

Other important issues are that this protocol proposes a double-blind study and the randomization will be performed in blocks.

Self-assessment has become an important tool to evaluate the results of aesthetic procedures,^[38]^ facial changes resulting from aging can impact the quality of life, self-esteem, social relationships, and activities of individuals. Thus, preventive and corrective interventions are closely related not only to health but also to psychosocial well-being.^[8,9]^ In this sense, the FACE-Q questionnaire, validated for the Portuguese language, will be used to verify the level of satisfaction after treatment.

It is known that most studies that evaluate the face are based on subjective measures (patient satisfaction and photos) or using skin biopsy.^[20]^ The evaluation methods used in this research, are noninvasive, and double-blind and aim to evaluate a set of variables that can be affected or improved by PBM as a result of the frequency of application in the groups. Face photographs will be evaluated by 3 plastic surgeons using the wrinkle scale.^[29]^ These experts will be calibrated for this assessment and must achieve a rate of agreement among peers above 70%. The evaluation of the images by the Image J software, performed by a researcher not involved in this study, aims to bring quantitative data for visual analysis. The researcher will quantify in pixels the wrinkles of interest before and after treatment. The evaluation of wrinkles in plaster casts by OCT is a reliable and accurate method and there are few data in the literature that provide this type of evaluation. Therefore, in addition to contributing as an evaluation in this protocol, it could become a measurement tool in the area of aesthetics.

In summary, this clinical trial aims to contribute to clarifying the role of PBM application frequency in improving the appearance of the facial skin of menopausal women using qualitative and quantitative methodologies.

## Author contributions

**Conceptualization:** Erick Frank Bragato, Jefferson André Pires, Adriana Fernandes Paisano, Kristianne Porta Santos Fernandes.

**Data curation:** Erick Frank Bragato.

**Formal analysis:** Erick Frank Bragato.

**Funding acquisition:** Erick Frank Bragato, Jefferson André Pires, Marcos Momolli, Marina Bertoni Guerra.

**Investigation:** Erick Frank Bragato, Jefferson André Pires, Marcos Momolli.

**Methodology:** Erick Frank Bragato, Adriana Fernandes Paisano, Raquel Agnelli Mesquita Ferrari, Kristianne Porta Santos Fernandes.

**Project administration:** Erick Frank Bragato, Marina Bertoni Guerra, Adriana Fernandes Paisano, Kristianne Porta Santos Fernandes.

**Resources:** Erick Frank Bragato, Adriana Fernandes Paisano, Raquel Agnelli Mesquita Ferrari, Lara Jansiski Motta, Kristianne Porta Santos Fernandes.

**Software:** Erick Frank Bragato.

**Supervision:** Erick Frank Bragato, Sandra Kalil Bussadori, Lara Jansiski Motta, Kristianne Porta Santos Fernandes.

**Validation:** Erick Frank Bragato, Sandra Kalil Bussadori, Kristianne Porta Santos Fernandes.

**Visualization:** Erick Frank Bragato, Raquel Agnelli Mesquita Ferrari, Sandra Kalil Bussadori, Lara Jansiski Motta, Kristianne Porta Santos Fernandes.

**Writing – original draft:** Erick Frank Bragato.

**Writing – review & editing:** Erick Frank Bragato.
